# Understanding the epidemiology and perceived efficacy of cannabis use in patients with chronic musculoskeletal pain

**DOI:** 10.1186/s42238-024-00231-1

**Published:** 2024-07-03

**Authors:** Timothy Leroux, Prabjit Ajrawat, Kala Sundararajan, Naomi Maldonado-Rodriguez, Bheeshma Ravi, Rajiv Gandhi, Raja Rampersaud, Christian Veillette, Nizar Mahomed, Hance Clarke

**Affiliations:** 1 The Arthritis Program, University Health Network, 399 Bathurst St, East Wing, 1st Floor, Room 1-436, Toronto, Ontario M5T 2S8 Canada; 2 Department of Anesthesia and Pain Management, Toronto General Hospital, University Health Network, Toronto, Ontario Canada; 3 Sunnybrook Health Sciences Centre, Toronto, Ontario Canada

**Keywords:** Cannabinoids, Cannabis, Chronic musculoskeletal pain, Musculoskeletal, Pain, Orthopaedics

## Abstract

**Background:**

The belief that cannabis has analgesic and anti-inflammatory properties continues to attract patients with chronic musculoskeletal (MSK) pain towards its use. However, the role that cannabis will play in the management of chronic MSK pain remains to be determined. This study examined 1) the rate, patterns of use, and self-reported efficacy of cannabis use among patients with chronic MSK pain and 2) the interest and potential barriers to cannabis use among patients with chronic MSK pain not currently using cannabis.

**Methods:**

Self-reported cannabis use and perceived efficacy were prospectively collected from chronic MSK pain patients presenting to the Orthopaedic Clinic at the University Health Network, Toronto, Canada. The primary dependent variable was current or past use of cannabis to manage chronic MSK pain; bivariate and multivariable logistic regression were used to identify patient characteristics independently associated with this outcome. Secondary outcomes were summarized descriptively, including self-perceived efficacy among cannabis users, and interest as well as barriers to cannabis use among cannabis non-users.

**Results:**

The sample included 629 patients presenting with chronic MSK pain (mean age: 56±15.7 years; 56% female). Overall, 144 (23%) reported past or present cannabis use to manage their MSK pain, with 63.7% perceiving cannabis as very or somewhat effective and 26.6% considering it as slightly effective. The strongest predictor of cannabis use in this study population was a history of recreational cannabis use (OR 12.7, *p*<0.001). Among cannabis non-users (*N*=489), 65% expressed interest in using cannabis to manage their chronic MSK pain, but common barriers to use included lack of knowledge regarding access, use and evidence, and stigma.

**Conclusions:**

One in five patients presenting to an orthopaedic surgeon with chronic MSK pain are using or have used cannabis with the specific intent to manage their pain, and most report it to be effective. Among non-users, two-thirds reported an interest in using cannabis to manage their MSK pain, but common barriers to use existed. Future double-blind placebo-controlled trials are required to understand if this reported efficacy is accurate, and what role, if any, cannabis may play in the management of chronic MSK pain.

**Supplementary Information:**

The online version contains supplementary material available at 10.1186/s42238-024-00231-1.

## Introduction

Musculoskeletal (MSK) disorders are the most common cause of functional disability and chronic pain worldwide (Pinto et al. 2017; Sundstrup et al. 2016). Osteoarthritis (OA) is a common cause of MSK related pain and disability with persistent pain typically being the catalyst for seeking medical intervention (Pinto et al. 2017; Yelin et al. 2016). As the global population ages, the burden of chronic MSK pain is expected to rise, substantially increasing societal costs and healthcare resource utilization (Pinto et al. 2017; Yelin et al. 2016). Current pharmacological (NSAIDs, COX-2 inhibitors, acetaminophen, serotonin-norepinephrine reuptake inhibitors, anticonvulsants, opioids, gabapentinoids) and non-pharmacological (physiotherapy, acupuncture, mindfulness/psychotherapy, transcutaneous electrical nerve stimulation therapy, etc.) approaches to manage chronic MSK pain have at best delivered modest efficacy and can often cause harmful adverse events. Given the unreliability of these therapies, it is not surprising that many chronic MSK patients often seek non-opioid and non-surgical alternatives for pain management (Boehnke et al. 2019).

Recreational cannabis use is now legal in several countries and there is a trend towards further legalization globally. More recently, the use of cannabis as an analgesic, immunomodulatory, and anti-inflammatory agent for patients with chronic MSK pain has become relevant (Ajrawat et al. 2022; Meng et al. 2021). Studies have shown the endocannabinoid system to be expressed in various cell types within human joints, including synovial cells, chondrocytes, and bone cells (Dunn et al. 2016; Porta et al. 2014). Clinical cannabinoid research to date has largely focused on neuropathic pain or chronic non-cancer pain (CNCP), with previous studies indicating that 10-15% of chronic pain patients use cannabis to manage their pain (Piper et al. 2017; Haroutounian et al. 2016; Hazekamp et al. 2013; Troutt and DiDonato 2015). Prior systematic reviews have demonstrated cannabis’ effectiveness in managing chronic pain and potentially reducing opioid consumption (Troutt and DiDonato 2015; Whiting et al. 2015). A study observing 757 patients who used a combination of dried or oil-based cannabis formulations revealed that, after 12 months of follow-up, the percentage of individuals reporting opioid use decreased by approximately half, dropping from 40.8% to 23.9% (Meng et al. 2021). Another study investigated chronic osteoarthritis (OA) patients, most of whom were using either cannabidiol (CBD) or tetrahydrocannabinol (THC) products in a sublingual tincture form. The findings revealed lower pain scores, improved quality of life, and a decrease in morphine milligram equivalents (MME) among these patients with 37.5% of participants achieving 0 MME/day at the 6-month follow-up (Renslo et al. 2022). A third cross-sectional study investigated the use CBD on arthritic symptoms among OA, rheumatoid, and other autoimmune arthritis patients. The authors reported that while all groups lowered their medications use and improved their arthritic symptoms, the OA group had the greatest improvements in physical function and pain with CBD use (Frane et al. 2022). Although past studies have been promising, these effects are still poorly investigated in patients experiencing chronic MSK pain, limiting the generalizability to this large patient population.

Therefore, the purpose of the present study was 1) to determine the patient reported prevalence rate, patterns of use, and perceived efficacy of cannabis use among patients with chronic MSK pain (i.e., pain on most days for at least three months duration) referred for orthopaedic consultation and 2) to identify the interest and potential barriers to cannabis use among patients with chronic MSK pain not currently using cannabis.

## Methods

### Study design and setting

In this cross-sectional study, we administered an anonymous electronic survey regarding cannabis use to consecutive patients presenting with chronic MSK pain to the Orthopaedic clinic at the University Health Network (UHN), Toronto, Canada. All potentially eligible patients visiting the clinic between November 1, 2018 and April 30, 2019 were informed of the study by a designated member of the patient’s circle of care. If the patient expressed interest, a research assistant obtained electronic written consent via a tablet and confirmed the patient’s eligibility, then invited the patient to complete the tablet-based survey (Supplementary Tables 1-3). The study was approved by the UHN Research Ethics Board (REB Number: 18-5716).

### Participants

Patients with chronic MSK pain who were visiting the Orthopaedic Clinic at Toronto Western Hospital, University Health Network for a first-time consultation with an orthopaedic surgeon were included. Patients were deemed eligible to participate if they met the following inclusion criteria: (1) were ≥18 years of age, (2) able to speak and comprehend English, (3) and reported having chronic MSK pain, defined as muscle, tendon, bone or joint pain on most days for at least three months duration. Patients were excluded if they had sustained an acute MSK soft-tissue injury or fracture in the past 6 months, or if they had undergone surgery in the past 6 months.

### Development of survey

The survey was designed to collect data on patient demographics, current health status and pharmaceutical pain management, characteristics of MSK pain, and cannabis use. A team of surgeons and clinical experts in the field self-developed the survey. To create it, we utilized questions from the Longitudinal Evaluation in the Arthritis Program (LEAP) study as a foundation and then developed questions specific to cannabis use based on the available literature (Whiting et al. 2015; Walsh et al. 2013; Power et al. 2019). Although this survey was not internally or externally validated, 3-4 research personnel reviewed the questionnaire on iPads to ensure clarity, coherence, and technical functionality before administering it to patients.

All respondents completed the demographics, health status, and cannabis use sections of the survey (Supplementary Table 1). Respondents who reported current or past use of cannabis to manage MSK pain (“cannabis users”) also answered more detailed questions regarding their duration and frequency of cannabis use, strain/type consumed, monthly cost, mode of administration, provenance, cannabis-related side-effects, and reason for initiating use (Supplementary Table 2). Cannabis users were also asked to report their perception of cannabis’s effectiveness for alleviating chronic MSK pain, its effectiveness compared to other prescription medication(s) and change in use of other medications since starting to use cannabis. Respondents who reported never using cannabis to manage MSK pain (“cannabis non-users”) were asked to rate their interest in using cannabis to manage chronic MSK pain, as well as potential barriers to using cannabis for this purpose (Supplementary Table 3).

### Outcomes

The primary outcome of this study was current or past cannabis use for chronic MSK pain management, defined as a response of “yes” on the question “Have you ever used cannabis to manage your musculoskeletal pain (which includes muscle, tendon, bone or joint pain)?” Secondary outcomes were stratified based on reported cannabis use for the purposes of managing chronic MSK pain. Among cannabis users, secondary outcomes included patterns of cannabis use, self-perceived effectiveness, and self-reported changes in pain medications with cannabis use. Among cannabis non-users, secondary outcomes included interest in using cannabis for the management of chronic MSK pain and barriers to its use.

### Covariates

Patient demographics (age, gender, ethnic group, education, labour force participation, household income, and body-mass index), comorbid conditions, use of other pain medications (over-the-counter analgesics, prescription non-steroidal anti-inflammatory drugs, muscle relaxants, narcotic drugs, antidepressant drugs, and neuroleptic drugs), pain characteristics (bodily region for which the respondent is seeking care, number of painful bodily regions, and duration of pain), history of chronic pain specialist consultation, and recreational cannabis use were assessed as potential predictors of cannabis use for the management of chronic MSK pain.

### Data extraction and collection

Participants entered survey responses directly into an electronic database via a tablet-based survey application. Analyses were carried out in R Statistical Software version 3.6.1 (Rdct and R: A, 2011). 

### Statistical analysis

Patient characteristics, patterns of cannabis use, and its self-perceived effectiveness and side effects were summarized descriptively; continuous measures were summarized with means and standard deviations, and categorical measures with counts and percentages. Bivariate analysis was conducted comparing cannabis users (patients reporting current or past cannabis use to manage their chronic MSK pain) versus non-users (patients who reported never using cannabis to manage MSK pain), using the Mann-Whitney test for continuous variables and Fisher’s exact test for categorical variables. We expected that approximately 5% of patients who visit the orthopaedic clinic will use cannabis, based on data from Ste-Marie and colleagues (Liu et al. 2019). The minimum sample size is given by n = (z/m)^2 x p(1-p), where Z is the z-score corresponding to the desired confidence level, m is the desired level of precision, and p is the expected proportion of patients who use cannabis for therapeutic purposes. With Z = 1.96, m = 0.05, and *p* = 0.05, the minimum required sample size is 73 participants.

*P*-values from bivariate analysis were adjusted to control false discovery rate (FDR) (Benjamini and Hochberg 1995). To identify factors independently associated with medical cannabis use, factors found to be significantly associated with cannabis use in the bivariate analysis (*p* < 0.05) were entered in a multivariable logistic regression model of cannabis use along with age, gender, and ethnicity. Model fit was assessed with the area under the receiver operator curve and McFadden’s pseudo-R^2^. Results of the multivariable logistic regression were reported as Odds Ratios (OR) with 95% confidence intervals (CI). After reviewing bivariate analysis results, the following terms were entered into the model: age; gender; ethnicity (coded as White vs. non-White); labour force participation (coded as working/looking for work/short-term disability, retired/homemaker/student/other, or not looking for work/long-term disability); self-reported depression; number of comorbid conditions reported, excluding depression and MSK pain conditions (coded as none, 1-2 conditions, or 3+ conditions); use of narcotic medication (coded as never vs. sometimes/daily); ever visiting a pain clinic or specialist; bodily region for which the respondent is seeking treatment (coded as upper extremity, lower extremity, or spine); total number of painful bodily areas (coded as 1, 2, 3, or 4+ areas), duration of MSK pain (coded as 0-1 years, 1-5 years, or 5-10 years), and history of recreational cannabis use (coded as never vs. current/former use).

## Results

### Patient characteristics

**Fig. 1:**
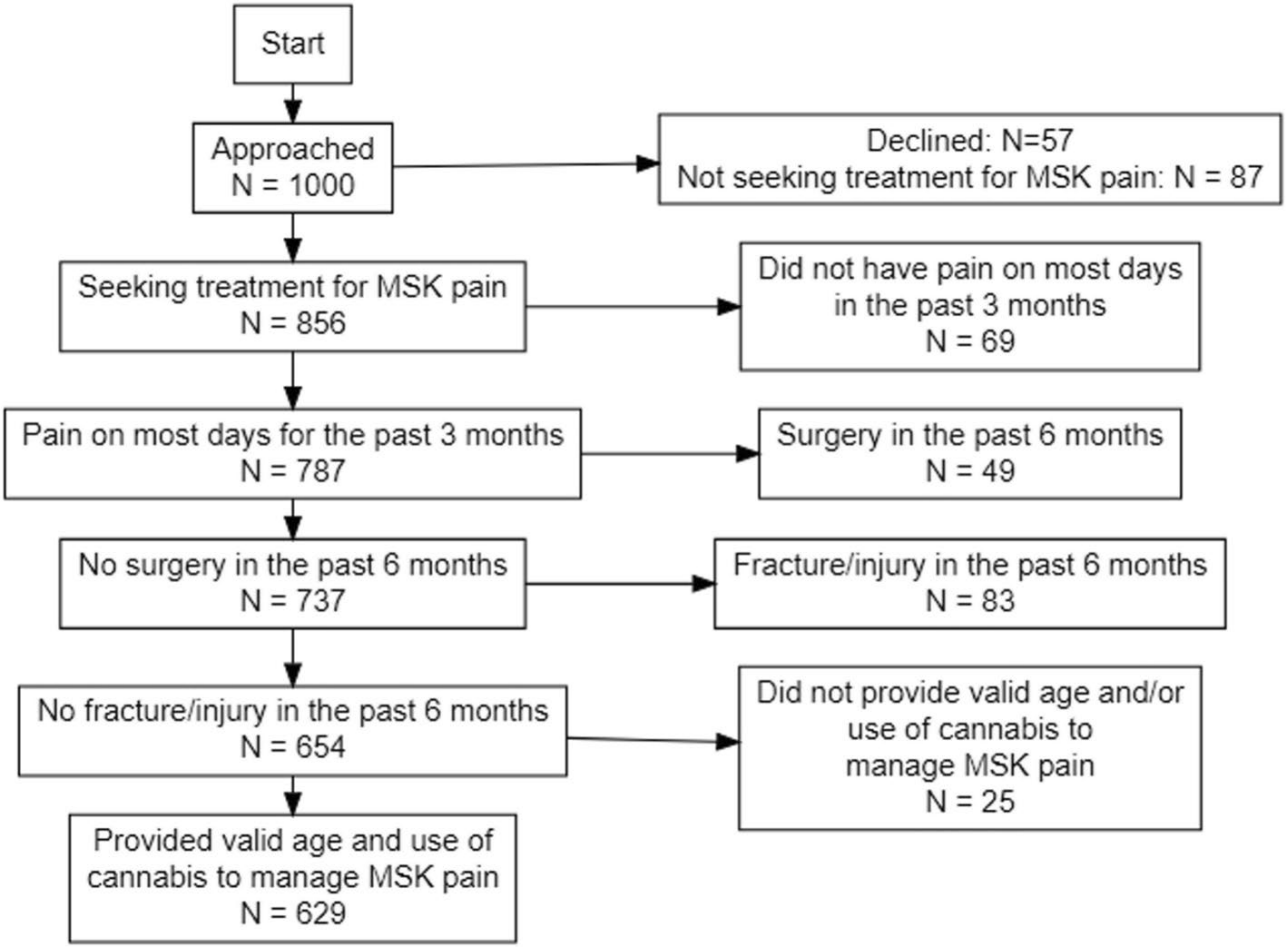
Sample flow diagram; MSK: musculoskeletal

**Fig. 2:**
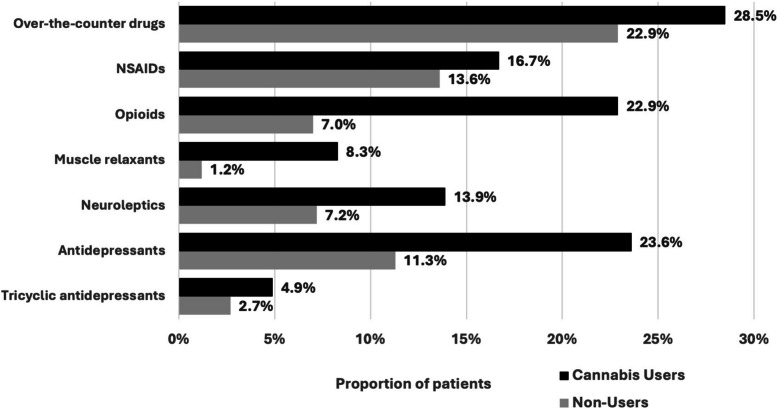
Distribution of daily pain medication use among patients with chronic musculoskeletal pain presenting to the orthopaedic clinic; NSAIDS: Non-steroidal anti-inflammatory drugs

One thousand patients presented to the orthopaedic clinic and were approached for study enrolment, of which 629 met the inclusion criteria and were included (Fig. 1). A total of 144 (23%) patients reported any (previous or current) cannabis use with the specific intention of managing their chronic MSK pain, of which 72% (*N*=104) were currently using cannabis to manage their pain. Cannabis users had a mean age of 50.88 ± 15.31 years and 51% (*N*=73) were females (Table 1). In comparison to cannabis non-users, cannabis users reported higher use of daily pain medications (Fig. 2).

**Table 1:** Patient demographics

**Measure**	**Response**	**Non-users** **(*N*** ** = 485)**	**Cannabis users** **(*N*** ** = 144)**	***p***
Age (y)	—	56.99 ± 15.55	50.88 ± 15.31	**<0.001**
Sex	Female	57.1% (277)	50.7% (73)	0.30
	Male	42.9% (208)	49.3% (71)	
Ethnicity	White	72% (349)	79.9% (115)	0.17
	Black	5.2% (25)	2.1% (3)	
	Latin/Central/South American	2.7% (13)	1.4% (2)	
	West Asian/Arab	2.7% (13)	0.7% (1)	
	South Asian	5.4% (26)	2.8% (4)	
	East Asian	2.7% (13)	0.7% (1)	
	Southeast Asian	0.4% (2)	0% (0)	
	Indigenous Peoples of North America	0.2% (1)	0% (0)	
	Other	3.3% (16)	3.5% (5)	
	Prefer not to answer	2.7% (13)	1.4% (2)	
	Multiple ethnic groups	2.9% (14)	7.6% (11)	
Highest education attained	Less than secondary school	5.8% (28)	6.9% (10)	0.17
	Secondary school diploma	17.7% (86)	16.7% (24)	
	Post-secondary certificate or diploma	24.3% (118)	33.3% (48)	
	Bachelor's degree	29.1% (141)	27.8% (40)	
	Degree in medicine, dentistry, law, etc.	4.7% (23)	6.2% (9)	
	Master's degree	13.2% (64)	7.6% (11)	
	Doctoral degree	2.7% (13)	0% (0)	
	Other	2.5% (12)	1.4% (2)	
Employment status	Employed full-time	39.2% (190)	38.2% (55)	**0.002**
	Employed part-time	10.1% (49)	10.4% (15)	
	Leave of absence/short-term disability	1% (5)	2.1% (3)	
	Long-term disability	4.7% (23)	13.9% (20)	
	Unemployed - looking for work	2.1% (10)	2.1% (3)	
	Not employed - not looking for work	1.2% (6)	5.6% (8)	
	Student	3.7% (18)	1.4% (2)	
	Homemaker	2.7% (13)	2.8% (4)	
	Retired	32% (155)	18.8% (27)	
	Other	3.3% (16)	4.9% (7)	
Annual pre-tax household income	<$30,000	11.5% (56)	17.4% (25)	0.12
	$30,000 - $44,999	9.1% (44)	4.9% (7)	
	$45,000 - $59,999	8.7% (42)	11.8% (17)	
	$60,000 - $100,000	19.8% (96)	11.8% (17)	
	≥$100,000	29.7% (144)	32.6% (47)	
	Prefer not to answer	21.2% (103)	21.5% (31)	
Body-mass index (BMI, kg/m2)	—	28.27 ± 7.13 (*N* = 469)	27.68 ± 7.19 (*N* = 139)	0.50
Comorbid condition(s)	High blood pressure	31.8% (154)	16.7% (24)	**0.002**
	Lung disease (e.g. asthma, COPD)	9.3% (45)	10.4% (15)	0.81
	Diabetes	6.8% (33)	6.9% (10)	1.00
	Ulcer or stomach disease	6.8% (33)	7.6% (11)	0.79
	Kidney disease	2.7% (13)	0.7% (1)	0.33
	Liver disease	3.3% (16)	2.1% (3)	0.67
	Anaemia/blood disease	5.8% (28)	6.2% (9)	0.88
	Cancer	6.4% (31)	8.3% (12)	0.58
	Depression	13.8% (67)	31.2% (45)	**<0.001**
	Osteoarthritis	51.5% (250)	54.9% (79)	0.61
	Back pain	48.5% (235)	76.4% (110)	**<0.001**
	Rheumatoid arthritis	13% (63)	16.7% (24)	0.42
	Heart attack/coronary artery disease	3.3% (16)	4.9% (7)	0.58
	Heart failure	0.2% (1)	0.7% (1)	0.55
	Stroke	1.9% (9)	1.4% (2)	1.00
	High cholesterol	21.2% (103)	14.6% (21)	0.17
	Thyroid problems	15.1% (73)	12.5% (18)	0.61
	Sleep apnea	15.9% (77)	16.7% (24)	0.85
	Dementia	3.3% (16)	2.1% (3)	0.67
	Chronic neck pain	16.9% (82)	35.4% (51)	**<0.001**
	Migraine headaches	15.7% (76)	22.2% (32)	0.15
	Chronic pelvic pain	4.5% (22)	20.1% (29)	**<0.001**
	Fibromyalgia	5.8% (28)	11.1% (16)	0.09
Number of conditions	—	3.03 ± 2.29	3.8 ± 2.05	**<0.001**
Ever used cannabis recreationally	Yes, currently (past 3 months)	4.7% (23)	50% (72)	**0.001**
	Yes, in the past	20.4% (99)	27.8% (40)	
	Never used recreationally	74.8% (363)	22.2% (32)	

Bold *p*-values are statistically significant (*p* < 0.05)

^*^Values presented as mean ± standard deviation or percentage (count)

In the bivariate analysis comparing cannabis users to non-users (Tables 1 and 2), cannabis use was associated with younger age (*p* < 0.001) and employment status (*p*=0.002), with users more likely to report being unemployed or on long-term disability. Cannabis users were more likely to have multiple comorbid conditions (*p* = 0.002), including depression (*p* < 0.001), back pain (*p* < 0.001), chronic neck pain (*p* < 0.001), and chronic pelvic pain (*p* < 0.001). They also reported a larger number of painful bodily areas (*p* < 0.001), longer pain durations (*p* = 0.003), more often visited a pain clinic/specialist (*p* = 0.003), and more frequently used muscle relaxants (*p* = 0.002), opioids (*p* = 0.002), and antidepressants (*p* = 0.002). Cannabis users were also more likely to report recreational cannabis use (*p* = 0.001). There were no statistically significant differences between cannabis users and non-users in sex, ethnicity, educational attainment, or income.

**Table 2:** History of pain and medication consumption

**Measure**	**Response**	**Non-users** **(*N*** ** = 485)**	**Cannabis users** **(*N*** ** = 144)**	***p***
***Medication use***				
Over-the-counter drugs	Never	18.1% (88)	18.1% (26)	0.53
	Sometimes	59% (286)	53.5% (77)	
	Daily	22.9% (111)	28.5% (41)	
Prescription NSAIDs	Never	55.1% (267)	49.3% (71)	0.55
	Sometimes	31.3% (152)	34% (49)	
	Daily	13.6% (66)	16.7% (24)	
Muscle relaxants	Never	72.2% (350)	62.5% (90)	**0.002**
	Sometimes	26.6% (129)	29.2% (42)	
	Daily	1.2% (6)	8.3% (12)	
Narcotic/opioid pain medications	Never	75.7% (367)	53.5% (77)	**0.002**
	Sometimes	17.3% (84)	23.6% (34)	
	Daily	7% (34)	22.9% (33)	
Antidepressants	Never	84.5% (410)	71.5% (103)	**0.002**
	Sometimes	4.1% (20)	4.9% (7)	
	Daily	11.3% (55)	23.6% (34)	
Neuroleptics	Never	86.2% (418)	78.5% (113)	0.09
	Sometimes	6.6% (32)	7.6% (11)	
	Daily	7.2% (35)	13.9% (20)	
Tricyclic antidepressants (for nerve pain)	Never	95.5% (463)	88.9% (128)	**0.02**
	Sometimes	1.9% (9)	6.2% (9)	
	Daily	2.7% (13)	4.9% (7)	
***MSK pain***				
Body region for which respondent is seeking treatment	Upper extremity	33.8% (164)	25% (36)	**0.002**
	Lower extremity	45.6% (221)	32.6% (47)	
	Spine	20.6% (100)	42.4% (61)	
Specific part of the body for which respondent is seeking treatment	Shoulder(s)	21.9% (106)	13.2% (19)	**0.003**
	Elbow(s)	3.1% (15)	4.2% (6)	
	Hip(s)	2.5% (12)	3.5% (5)	
	Knee(s)	26.8% (130)	18.1% (26)	
	Foot/feet	16.3% (79)	11.1% (16)	
	Ankle(s)	8% (39)	6.9% (10)	
	Wrist(s)	0.4% (2)	0% (0)	
	Hand(s)	0.4% (2)	0.7% (1)	
	Neck	2.9% (14)	5.6% (8)	
	Mid back	4.1% (20)	6.9% (10)	
	Low back	13.6% (66)	29.9% (43)	
Total number of painful areas reported	—	2.96 ± 1.99	3.81 ± 2.31	**<0.001**
Ever visited a pain clinic/pain specialist	Yes, currently	18.3% (85)	33.1% (47)	**0.003**
	Yes, in the past	19.1% (89)	19.7% (28)	
	Never	62.6% (291)	47.2% (67)	
	Not reported	— (20)	— (2)	
For how long have you had MSK pain?	3-6 months	9.9% (48)	3.5% (5)	**0.003**
	6-12 months	12.6% (61)	4.9% (7)	
	1-2 years	16.4% (79)	11.1% (16)	
	2-5 years	20.3% (98)	20.8% (30)	
	5-10 years	16.6% (80)	25% (36)	
	≥10 years	24.2% (117)	34.7% (50)	
	Not reported	— (2)	0% (0)	

Bold *p*-values are statistically significant (*p* < 0.05)

^*^Values presented as mean ± standard deviation or percentage (count)

### Patterns of cannabis use

A descriptive summary of cannabis users’ survey responses is presented in Table 3. Twenty-seven percent reported using cannabis for two or more years, and 25% for less than six weeks. Most patients used cannabis daily (59%). Thirty-nine percent of cannabis users reported spending at least $100 per month on cannabis products (Table 3).

**Table 3:** Patterns of use among current and past users of cannabis to manage MSK pain (*N*=144)

**Survey item**	**Response**	**% (count)**
Are you currently (within the last 3 months) using cannabis to manage your MSK pain?	Yes	72.2% (104)
	No	27.8% (40)
Do you intend to use cannabis to manage your MSK pain in the future?	Yes	82.5% (118)
	No	17.5% (25)
	Not reported	— (1)
How long have or did you use cannabis to manage your MSK pain?	<6 weeks	24.6% (35)
	6 weeks to 3 months	12.7% (18)
	3 to 6 months	10.6% (15)
	6 months to 1 year	12% (17)
	1 to 2 years	13.4% (19)
	≥2 years	26.8% (38)
	Not reported	— (2)
What strain/type of cannabis do or did you use? (select all that apply)	High-tetrahydrocannabinol (THC) strain	13.3% (19)
	Cannabidiol (CBD)	38.5% (55)
	Nabilone	0.7% (1)
	Levonantradol	0% (0)
	Dronabinol	0% (0)
	Nabiximols	0% (0)
	A mix of the above	19.6% (28)
	Unspecified/conventional cannabis	4.9% (7)
	I am not sure	23.1% (33)
	Not reported	— (1)
How often do or did you use cannabis for the management of MSK pain?	Daily	59.4% (85)
	Weekly	18.9% (27)
	Monthly	6.3% (9)
	Less than once a month	15.4% (22)
	Not reported	— (1)
How much do or did you spend monthly on cannabis to manage MSK pain?	<$50	30.1% (43)
	$50-100	30.8% (44)
	$100-200	24.5% (35)
	$200-400	11.9% (17)
	≥$400	2.8% (4)
	Not reported	— (1)
Why did you start using cannabis for MSK pain? (select all that apply)	Advised/recommended by an MD	25.7% (37)
	Advised by a health professional other than MD	6.2% (9)
	Advised by family member, friend, or another individual who is not a health professional	25.7% (37)
	My pain was insufficiently controlled using other medications and/or treatments	17.4% (25)
	I was interested in managing pain without conventional medications and/or treatments	24.3% (35)
	I wanted to avoid side-effect(s) that I experienced using other medications and/or treatments	20.1% (29)
	Other	6.2% (9)
What is or was your preferred mode of cannabis use? (select all that apply)	Smoking	36.4% (52)
	Vaporizing	31.5% (45)
	Oil	56.6% (81)
	Tinctures	6.3% (9)
	Capsules	9.8% (14)
	Edibles	22.4% (32)
	Topical application	9.8% (14)
	Patch formulation	0.7% (1)
	Other method	0.7% (1)
	Not reported	— (1)
Where do or did you acquire your cannabis? (select all that apply)	Friend/relative/someone I know	33.1% (47)
	Cannabis dispensary/Compassionate club	43% (61)
	Dealer/on the street	4.2% (6)
	Homegrown, without license	6.3% (9)
	Homegrown, with license	1.4% (2)
	Health Canada licensed provider	33.8% (48)
	Not reported	— (2)
In your opinion, how effective was or has cannabis been at controlling your MSK pain?	Very effective	25.9% (37)
	Somewhat effective	37.8% (54)
	Slightly effective	23.1% (33)
	Not effective	13.3% (19)
	Not reported	— (1)
In your opinion, how effective has cannabis been in controlling your MSK pain compared to prescription medication?	Much more effective	17.6% (25)
	Somewhat more effective	39.4% (56)
	No difference	21.8% (31)
	Somewhat less effective	9.9% (14)
	Much less effective	11.3% (16)
	Not reported	— (2)
Has your use of other pain medications changed since you started using cannabis?	Increased use of all other pain medications	0.7% (1)
	Use of all other pain medications unchanged	57.6% (80)
	Decreased use of all other pain medications	39.6% (55)
	Multiple changes reported	2.2% (3)
	Not reported	— (5)
Side-effects/negative effects from taking cannabis for MSK pain (select all that apply)	Dizziness	8.7% (12)
	Dry mouth	42.8% (59)
	Nausea	5.8% (8)
	Fatigue	22.5% (31)
	Lack of motivation	14.5% (20)
	Depression	2.9% (4)
	Vomiting	0.7% (1)
	Weight gain	5.8% (8)
	Euphoria	9.4% (13)
	Other	5.1% (7)
	None of the above	38.4% (53)
	Not reported	— (6)
Have you ever used cannabis to treat a symptom/condition other than MSK pain?	No	49.3% (70)
	Yes, MSK pain first	22.5% (32)
	Yes, other condition(s) first	28.2% (40)
	Not reported	— (2)
Symptoms/conditions other than MSK pain treated using cannabis (select all that apply)	Nausea	16.7% (23)
	Headache	18.1% (25)
	Sleep disturbances	44.2% (61)
	Appetite/weight	9.4% (13)
	Anxiety	26.1% (36)
	Depression	15.2% (21)
	Post-traumatic stress disorder	8% (11)
	Other	5.1% (7)
	None of the above	42.8% (59)
	Not reported	— (6)

Among cannabis users, CBD was the most commonly used cannabinoid (39%), followed by 20% of users consuming a hybrid of various cannabinoids. Twenty-three percent of cannabis users were unaware of their cannabis composition. The most frequent modes of administration among cannabis users were the ingestion of oils (57%), smoking (36%), and vaporizing (32%).

Twenty-six percent of users reported that they started using cannabis for pain management on the recommendation of a physician, and an equal proportion reported that a friend or relative advised them to use cannabis to manage pain (the options were not mutually exclusive). Many users reported getting cannabis from more than one source, including a dispensary or compassion club (43% of users); a Health Canada licensed provider (34%), and from a friend or relative (33%, Table 3).

### Perceived effectiveness

The majority of cannabis users noted effective pain management from cannabis, with 63.7% describing it as very or somewhat effective and 26.6% indicating it as slightly effective (Table 3). Fifty-seven percent of users indicated cannabis to be more effective than other analgesic medications at managing their chronic MSK pain (Table 3). Forty percent of users reported that their use of other analgesic medication had decreased since starting to use cannabis, while 58% of cannabis users indicated no change in their analgesic medication usage since initiating cannabis (Table 3).

Commonly reported cannabis-related side effects included dry mouth (43%), fatigue (23%), and a lack of motivation (15%). Thirty-nine percent of cannabis users experienced no cannabis-related side-effects. Many users indicated cannabis’s effectiveness in treating other symptoms, primarily sleep disturbances (44%), anxiety (26%), and headaches (18%). Forty-three percent of cannabis users experienced no other symptomatic reliefs.

### Predictors of current or past use of cannabis for MSK pain

Results of the multivariable regression analysis are illustrated in Table 4. The most significant predictor of cannabis use was current or previous recreational cannabis usage (OR:12.72, *p* = < 0.001). Other significant independent predictors of cannabis use included self-reported depression (2.17, *p* = 0.01); opioid medication use (1.78, *p* = 0.03); history of pain clinic/specialist visits (1.93, *p* = 0.01); seeking treatment for spine pain versus lower extremity pain (2.14, *p* = 0.01); a greater number of painful bodily areas (2.28 for three [*p* = 0.04] and 2.57 for four or more areas [*p* = 0.01], versus one area); and increased duration of MSK pain (2.28 for one to five years [*p* = 0.04] and 2.8 for five to ten years [*p* = 0.01], versus less than one year). While controlling for these factors, age, gender, ethnicity, labour force participation and number of comorbid conditions were not significant predictors of cannabis use for MSK pain management.

**Table 4:** Multivariable logistic regression: predictors of cannabis use for MSK pain (*N*=626)

**Predictor**	**Term**	**OR [95% CI]**	***P***
**Age**	per 5 y	0.95 [0.87-1.03]	0.22
**Gender** (reference: female)	Male	1.29 [0.79-2.11]	0.30
**Ethnicity** (reference: White)	Non-white	0.84 [0.47-1.47]	0.54
**Labour force participation** (reference: working, looking for work, or short-term disability)	Retired, homemaker, student, or other	0.93 [0.52-1.67]	0.81
	Not looking for work or long-term disability	1.69 [0.76-3.72]	0.19
**Mental health**	Self-reported depression	2.17 [1.21-3.89]	**0.01**
**Comorbid conditions ex. depression, MSK-related** (reference: none reported)	1-2 conditions	1.22 [0.68-2.21]	0.51
	3+ conditions	0.89 [0.42-1.86]	0.75
**Opioid medication use** (reference: never)	Sometimes or daily	1.78 [1.05-3.04]	**0.03**
**Pain clinic/specialist visit**	Ever visited	1.93 [1.18-3.18]	**0.01**
**Body region for which respondent is seeking treatment** (reference: Lower extremity)	Upper extremity	0.91 [0.51-1.61]	0.74
	Spine	2.14 [1.19-3.88]	**0.01**
**Total number of painful areas of body, of 11** (reference: 1 area)	2	1.27 [0.59-2.76]	0.55
	3	2.28 [1.05-5.01]	**0.04**
	4 or more	2.57 [1.31-5.23]	**0.01**
**Duration of MSK pain** (reference: <1 year)	1-5 years	2.28 [1.04-5.27]	**0.04**
	5-10 years	2.8 [1.3-6.43]	**0.01**
**Recreational cannabis use** (reference: never used)	Current or former use	12.72 [7.54-22.25]	**<0.001**

*MSK* Musculoskeletal, *AUC* Area under the receiver-operator curve, *OR* Odds ratio, *CI* Confidence interval

^*^McFadden's pseudo-R^2^= 0.327, AUC = 0.871

### Non-cannabis users

**Fig. 3:**
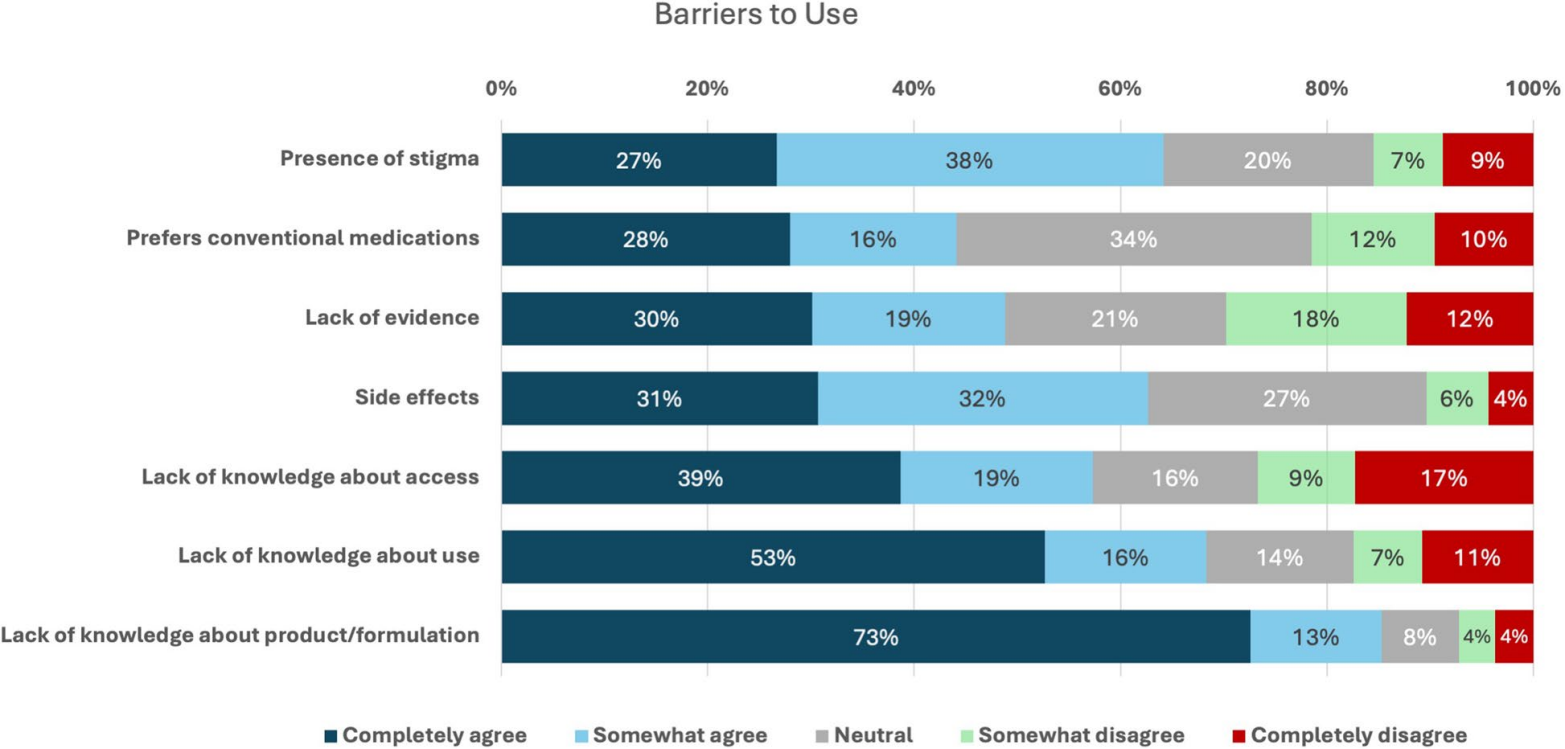
Non-cannabis users’ rating of factors influencing cannabis use for the management of chronic MSK pain

Sixty-five percent of cannabis non-users reported an interest in using cannabis for managing their chronic MSK pain. Given a list of potential barriers to using cannabis, the majority of non-users “completely” or “somewhat” agreed with many of the barriers, especially a lack of knowledge regarding cannabis formulations (85%), how to use cannabis for pain management (68%), and how to access cannabis for pain management (57%,); potential cannabis-related side effects (63%); and a perception of stigma (64%) (Fig. 3).

## Discussion

In this study, more than one in five patients presenting to an orthopaedic surgeon with chronic MSK pain had used or was currently using cannabis to manage their chronic MSK pain. Consistent with previous self-reported studies, the majority of these patients perceived cannabis to provide effective pain management (Ajrawat et al. 2022; Meng et al. 2021). More than half (57%) claimed cannabis to be more effective than other analgesic medications, and 40% reported decreasing their use of other analgesic medications since starting cannabis use. Only 26% of cannabis users reported that a physician recommended the use of cannabis to manage their chronic MSK pain, and 23% were unaware of the composition of the cannabis they were consuming. Among non-users, 65% expressed an interest in using cannabis for managing their chronic MSK pain but often reported that they lacked knowledge regarding efficacy, access, usage, and composition, as well as the associated stigma.

We found that 23% of chronic MSK pain patients have used or are using cannabis to manage their pain, of which the majority are current users (72%). This rate of cannabis use among chronic MSK pain patients in the present study is considerably higher than the cannabis use rates reported in past studies with similar patient populations. For instance, two different studies, one from Canada and another from the United States, indicated that 4% of patients undergoing orthopaedic procedures used cannabis (Liu et al. 2019; Medina et al. 2019). Similarly, a 2014 Canadian study reported that approximately 3% of rheumatology patients were cannabis users (Ste-Marie et al. 2016). Yet, a more recent study conducted in Canada revealed that 29% of individuals with upper extremity conditions were presently utilizing cannabis, primarily for the purpose of pain management (Greis et al. 2022). The difference in prevalence between the previous studies from 3-6 years ago and the current study is largely due to the legalization of cannabis in Canada in October 2018, resulting in potential changes in attitudes towards and acknowledgment of use and more permissive access to cannabis without stigma and/or legal implications. In fact, a study assessing data from the National Cannabis Survey (NCS) revealed that in the months preceding legalization, 19% of Canadian respondents intended to try cannabis or increase their cannabis use following legalization (Sandhu et al. 2019). Complementing this, a 2014 US survey found that 13.5% of people would consume cannabis more frequently if legalized (Cohn et al. 2017). Additionally, the anonymous self-reported nature of the current study may have also allowed patients to feel comfortable disclosing their cannabis, resulting in the higher rate we observed.

The strongest predictors of those who would use cannabis for managing their chronic MSK pain, were patients with a history of recreational cannabis use and the presence of long-term chronic pain. These findings are consistent with previous reports examining cannabis use in various patient populations (Liu et al. 2019; Medina et al. 2019; Sandhu et al. 2019; Bhashyam et al. 2019). It was found that previous recreational cannabis use was associated with a more than tenfold increase in the odds of using cannabis to manage chronic MSK pain. This was unsurprising as current cannabis use has been shown to strongly influence one’s perceptions of risk, stigma, and acceptability (Rudski 2014). Features consistent with the diagnosis of a chronic pain syndrome, such as frequent pain clinic visits and longer pain duration, were also strong predictors of cannabis use in the current cohort, suggesting that patients with chronic MSK pain are possibly unsatisfied with conventional treatments and seeking alternative pain therapies. Lastly, younger age was not a predictor of using cannabis for the management of chronic MSK pain, when controlling for factors such as opioid consumption, depression status, and the duration of pain. This finding is inconsistent with past studies which have indicated that the incidence of any cannabis use (medical or recreational) generally declines with increasing age (Rotermann and Page 2018; Sampasa-Kanyinga et al. 2018). Typically, it is assumed that younger age is associated with cannabis use, which is likely because the belief among younger adults is that cannabis use is socially acceptable, not addictive, and not harmful (Kandel 2002; Keyhani et al. 2018; Ware et al. 2003). However, there may be several potential reasons for this discrepancy. First, the current study’s cohort is older overall (mean age >50 years) and includes fewer young adults that would typically report a greater prevalence of use as compared to elderly patients. Second, this finding may suggest a growing acceptance among older patients regarding the role of cannabis use for the purpose of managing medical issues, which may be the result of the legalization in Canada, increasing societal acceptance, and improvements in accessibility of non-inhaled cannabis products.

In this study, over 85% of cannabis users perceived it to be effective in managing their chronic MSK pain and improving their sleep and anxiety-related symptoms. Several studies have also noted similar rates of perceived effectiveness in chronic pain patients (Heng et al. 2018; Miller and Miller 2017; Mucke et al. 2018). One study with 937 orthopaedic outpatients found that cannabis use was significantly associated with decreased pain intensity and better lower extremity activity scores (Medina et al. 2019). In a prospective observational study of chronic orthopaedic pain patients, medical cannabis use was associated with significant clinical improvements in VAS pain scores, global physical health and mental health, and quality of life within three months but plateaued at the 6 and 12 month follow-up periods (Greis et al. 2022). Furthermore, cannabinoids were found effective in all eight studies from a systematic review that evaluated chronic pain stemming from orthopedic etiologies (Vivace et al. 2021). Despite preliminary evidence suggesting cannabis’ pain management potential, most studies have short-term follow-ups with relatively small sample sizes. Moreover, significant heterogeneity exists among studies with regards to dosage, routes of administration, composition, frequencies, and patient populations, justifying the need for further investigations to elucidate the true efficacy of cannabis for the management of chronic MSK pain.

Several interesting observations were noted herein: a quarter of cannabis users had no knowledge of the current cannabis product they were consuming and only a third of users procured their cannabis from a Health Canada licensed provider. A study with upper extremity orthopaedic patients indicated that approximately 46% of patients felt more comfortable discussing their cannabis use with their physician after legalization (Sims et al. 2022). From a recent survey study, 86% of orthopaedic patients that were characterized as cannabis users stated that they would be willing to stop consuming cannabis if their surgeon stated it would adversely impact their surgery (Carney et al. 2020). These observations highlight the dire need for improved oversight and regulation of the medicinal cannabis industry (Craft et al. 2020; Freeman and Lorenzetti 2020). In the present study, 23% of cannabis users were unaware of their cannabis composition. Greis and colleagues reported similar findings with 23-29% of orthopaedic patients being able to estimate the cannabis composition of their inhaled or oral cannabis products (Greis et al. 2022). In our study, the most frequent modes of administration among cannabis users were the ingestion of oils (57%), smoking (36%), and vaporizing (32%). Similar to our results, a previous report indicated that most orthopaedic patients preferred oral or sublingual administration of cannabis followed by inhalation to manage their chronic pain (Greis et al. 2022). In contrast, Carney et al reported that smoking was the most common mode of administration, followed by edible products, and vaporizing (Carney et al. 2020). Although most patients in the present study reported cannabis to be effective, the varying compositions, dosages, frequencies, and routes of administration, suggests that placebo may mitigate some of the perceived efficacy. As such, there is a need for large scale observational studies where the dosages and route of administration are standardized. This could then lead to meaningful placebo-controlled comparative studies in similar patient populations to determine the effectiveness on symptom management.

Historically, common first-line pharmacologic treatments such as nonsteroidal anti-inflammatory drugs (NSAIDs) have been prescribed to alleviate chronic pain. However, NSAIDs have been associated with intolerance and serious adverse events in some patients including upper gastrointestinal bleeding or perforation (Bjordal et al. 2004; Langman et al. 1994). Opioids have also been prescribed to manage chronic MSK pain when conventional treatments have failed, so it is not surprising that individuals with chronic MSK pain are amongst the highest prescription opioid users (Ashaye et al. 2018; Manchikanti et al. 2012). However, long-term opioid use has been associated with greater pain intensity, poorer outcomes, and unintendedly increases the risk of developing an opioid use disorder, diversion, and fatal overdose (Bot et al. 2014; Dunn et al. 2010; Koehler et al. 2018; Noble et al. 2010). Cannabis has been suggested as a potentially safer analgesic therapy. In the present study, approximately 40% of our patients reported that their analgesic medications were reduced after initiating cannabis. A similarly designed study by Sims and colleagues with reported that 51% of orthopaedic patients felt that cannabis was safer than their prescription analgesics. (Sims et al. 2022) Further, Greis et al reported that 31% of chronic orthopaedic pain patients discontinued benzodiazepines, 73% either ceased or decreased opioid consumption, and noted a 23.4% reduction in 6-month total opioid prescription with cannabis use (Greis et al. 2022). Moreover, a systematic review evaluating the use of cannabinoids in orthopaedic patients found that the five of the seven studies noted an opioid sparring effect and two of the seven studies reported complete cessation of opioid use at the 6 to 12 month follow-up period (Vivace et al. 2021). Prior surveys of American and Canadian cannabis users reported that substituting cannabis for opioids resulted in improved pain management, decreased adverse effects, and eased opioid withdrawal symptoms. (Lucas and Walsh 2017; Vyas et al. 2018)

It is noteworthy that cannabis users exhibited a constellation of comorbid conditions, including a higher prevalence of depression and pain, an increased number of painful bodily areas, longer pain durations, and more frequent visits to pain clinics/specialists when compared to non-cannabis users. This observation prompts us to consider the possibility that cannabis use may have arisen as a response to elevated levels of pain and dissatisfaction with existing therapeutic modalities. It is possible that a significant proportion of cannabis users turned to cannabis to seek relief from their heightened pain burden, which appears refractory to conventional treatments. Furthermore, we observed that cannabis users, despite experiencing greater pain, tended to employ a broader array of medications, such as muscle relaxants, opioids, and antidepressants, in comparison to their non-cannabis-using counterparts. This may also highlight that cannabis may have been sought as an alternative means of pain management, especially in situations where previous therapies yielded suboptimal results.

In this cohort, approximately two-thirds of cannabis non-users expressed interest in using cannabis for managing their chronic MSK pain, but often reported that they lacked knowledge regarding efficacy, access, usage, and composition. Interestingly, stigma was not a primary concern for cannabis non-users as previously cited in the literature (Bottorff et al. 2013; Hathaway et al. 2011; Satterlund et al. 2015), possibly attributed to the recent cultural shifts with cannabis legalization and with the increasing number of cannabis products that do not need to be smoked for consumption (only one third of products consumed in this study were smoked), leading to greater social acceptability and normalization (Hathaway et al. 2011). Research indicates that individuals are learning about cannabis from acquaintances and the internet instead of healthcare professionals (Corroon and Phillips 2018), which is consistent with the findings of the current study whereby only a quarter of patients started using cannabis as a result of a physician’s recommendation. Although the general perception is that cannabis is safe, there is a potential for side effects and drug interactions of which patients need to be aware (Alsherbiny and Li 2018; Iffland and Grotenhermen 2017). However, a lack of confidence regarding their knowledge on cannabis safety and efficacy exists among physicians (Fitzcharles et al. 2014; Kondrad and Reid 2013), which may prevent them from either initiating or participating in discussions regarding medical cannabis use with their patients. As such, continuing education initiatives targeting physicians are important to ensure they are armed with basic, albeit necessary, information regarding medical cannabis.

With the increased legalization of cannabis across North America and the clear interest by MSK pain patients to use cannabis therapeutically, there is large knowledge gap to fill in understanding the role of cannabis, if any, in managing chronic MSK pain. Future investigations should aim to conduct high-quality multicentre double-blind placebo-controlled trials with larger sample sizes and longer durations to assess the clinical efficacy and the long-term adverse events of cannabis as a monotherapy and in conjunction with standard analgesics. Future studies should aim to determine the optimal dosage, dose-response effects, drug interactions, and the ideal composition and route of administration based on patient history and preference.

### Limitations

This study has several limitations. First, the survey utilized was not validated and the inability to compare characteristics of study participants with non-participants (i.e., those who declined or weren't eligible) limits the capacity to assess the external validity of the research. Second, the prevalence of cannabis use may be understated, given the stigma often associated with cannabis use and the use of self-reported data. However, the anonymous nature of the survey may have minimized this concern. Third, recall bias may have occurred with cannabis-related information, possibly affecting data accuracy. Additionally, the higher prevalence of comorbidities among cannabis users compared to non-users, may potential influence results and perceptions. Fourth, the survey only assessed the frequency and mode of administration and did not account for the dosage and precise composition of THC and CBD within the cannabis products being consumed. Fifth, the study was conducted in an academic, urban setting and limited to patients referred for Orthopaedic consultation, which limits the generalizability of these results to the broader MSK pain population. Finally, data collection started weeks following legalization at a single time interval, therefore the patient’s patterns and perceptions of cannabis efficacy and safety may change with increased awareness, social acceptability, and/or cannabis education since the time of the survey. We aim to repeat this work in the coming years to assess changes in use over time and collect validated patient reported outcome measures between cannabis users and non-users with chronic MSK pain, which will aim to improve upon some of the limitations identified herein.

## Conclusion

One in five patients presenting to an orthopaedic surgeon with chronic MSK pain are using or have used cannabis with the specific intent to manage their pain, and most report it to be effective. Among non-users, two-thirds reported an interest in using cannabis to manage their MSK pain, but common barriers to use existed. Future double-blind placebo-controlled trials are required to understand if this reported efficacy is accurate, and what role, if any, cannabis may play in the management of chronic MSK pain.

### Supplementary Information

**Supplementary Material 1.**

## Data Availability

All data generated or analysed during this study are included in this published article [and its supplementary information files].
