# Supplementary material for: Understanding the epidemiology and perceived efficacy of cannabis use in patients with chronic musculoskeletal pain
Source: J Cannabis Res. 2024 Jul 3;6:28. doi: 10.1186/s42238-024-00231-1 (PMC11220958; doi:10.1186/s42238-024-00231-1)
Supplement: Supplementary file 1 — Supplementary Material 1. [file 42238_2024_231_MOESM1_ESM.docx]

**APPENDICES:**

**Supplementary Table 1**

***Questions Assessing Inclusion***

Are you currently seeking treatment for musculoskeletal pain, which includes muscle, tendon, bone or joint pain?

Yes No If ‘NO’, patient is ineligible

If yes, have you experienced this pain most days for at least 3 months?

Yes No If ‘NO’, patient is ineligible

***Demographic Questions***

1. What is your age? _______
2. What is your sex?

Female Male Non-Binary

1. What statement best describes your current employment situation?

Employed full-time (e.g. 35+ hours per week)

Employed part-time (e.g. less than 35 hours per week)

Leave of absence/short-term disability

Long-term disability

Unemployed – looking for work

Not employed - not looking for work

Student

Homemaker

Retired

Other (please specify):_________________

1. What ethnic group do you most closely identify with? Please select all that apply.

White

Black

Latin/Central/South American

West Asian/Arab (e.g. Afghan, Iranian, Israeli, Turk, Lebanese)

South Asian (e.g. Indian, Pakistani, Bangladeshi)

East Asian (e.g. Chinese, Korean, Taiwanese)

Southeast Asian (e.g. Filipino, Indonesian, Thai, Vietnamese)

Indigenous Peoples of North America

Other (please specify): ___________________

Prefer Not to Answer

1. What is your annual household income (before taxes)?

Less than $30,000

$30,000 – $44,999

$45,000 - $59,999

$60,000 - $100,000

Greater than $100,000

Prefer Not to Answer

1. What is the highest degree or level of school you have completed? If currently enrolled, highest degree received?

Less than secondary or high school

Secondary/High school diploma, or equivalent

Post-secondary certificate or diploma (e.g. apprenticeship/trade, college, CEGEP, etc.)

University – Bachelor’s Degree

University – Degree in medicine, dentistry, veterinary medicine, law, optometry, etc.

University – Master’s Degree

University – Doctoral Degree

Other (please specify):______________­­­

1. What is your height? ______ cm or ______ inches
2. What is your weight? ______kg or _____ pounds
3. The following is a list of medical conditions. Please check ‘yes’ if you have been diagnosed with the condition or are receiving treatment for the condition, such as taking medication.

|  | *Do you have or are you receiving treatment for the condition?* | |
| --- | --- | --- |
|  | *Yes* | *No* |
| High blood pressure |  |  |
| Lung disease (e.g. asthma, COPD) |  |  |
| Diabetes |  |  |
| Ulcer or stomach disease |  |  |
| Kidney disease |  |  |
| Liver disease |  |  |
| Anemia or other blood disease |  |  |
| Cancer |  |  |
| Depression |  |  |
| Osteoarthritis/Degenerative arthritis |  |  |
| Back pain |  |  |
| Rheumatoid arthritis |  |  |
| Heart attack/Coronary artery disease |  |  |
| Heart failure |  |  |
| Stroke |  |  |
| High cholesterol |  |  |
| Thyroid problems |  |  |
| Sleep apnea |  |  |
| Dementia (memory loss, poor concentration) |  |  |
| Chronic neck pain |  |  |
| Migraine headaches |  |  |
| Chronic pelvic pain |  |  |
| Fibromyalgia |  |  |

1. Are you currently visiting a pain clinic or seeing a pain specialist, other than your visit here today?

Yes No

1. If ‘No’ to question 10, have you visited a pain clinic or seen a pain specialist in the past?

Yes No

1. Please indicate which of the following medications you are currently taking to manage your musculoskeletal pain (which includes muscle, tendon, bone or joint pain).

| Medication | Never | Sometimes | Daily |
| --- | --- | --- | --- |
| Over-the-Counter Drugs  Examples: Ibuprofen, Naproxen, Acetylsalicylic Acid, Acetaminophen | 🞎 | 🞎 | 🞎 |
| Prescription Non-Steroidal Anti-Inflammatory Drugs (NSAIDS) Examples: Diclofenac / Misoprostol, Celebrex, Diclofenac, Naproxen, Diclofenacm Meloxicam | 🞎 | 🞎 | 🞎 |
| Muscle Relaxants  Examples: Cyclobenzaprine Hydrochloride, Methocarbamol | 🞎 | 🞎 | 🞎 |
| Narcotic/Opioid Pain Medications  Examples: Meperidine, Morphine, Oxycodone Hydrochloride, Oxycodone Acetaminophen, Pentazocine, Acetaminophen-codeine | 🞎 | 🞎 | 🞎 |
| Anti-Depressants  Examples: Citalopram, Escitalopram, Duloxetine, Amitriptyline Hydrochloride, Paroxetine, Fluoxetine, Bupropion Hydrochloride, Sertraline | 🞎 | 🞎 | 🞎 |
| Neuroleptics (to calm nerve pain)  Examples: Pregabalin, Gabapentin, Clonazepam, Carbamazepine | 🞎 | 🞎 | 🞎 |
| Tricyclic Antidepressants (to calm nerve pain)  Examples: Amitryptiline, Nortryptiine | 🞎 | 🞎 | 🞎 |

***Visit-Specific Questions***

1. Which clinic are you visiting today?

Orthopaedic

Rheumatology

1. What part of the body are you currently seeking treatment for?

Shoulder(s)

Elbow(s)

Hip (s)

Knee(s)

Foot/Feet

Ankle(s)

Wrist(s)

Hand(s)

Neck

Mid-back

Low-Back

1. Please list all areas of the body that cause you pain? Check all that apply.

Shoulder(s)

Elbow(s)

Hip(s)

Knee(s)

Foot/Feet

Ankle(s)

Wrist(s)

Hand(s)

Neck

Mid-back

Lower-back

1. Is the primary reason for your visit today due to musculoskeletal pain (which includes muscle, tendon, bone or joint pain)?

Yes

No

1. For how long have you had musculoskeletal pain (which includes muscle, tendon, bone or joint pain)?

Most days for 3 months to less than 6 months

Most days for 6 months to less than 1 year

Most days for 1 year to less than 2 years

Most days for 2 years to less than 5 years

Most days for 5 years to less than 10 years

Most days for 10 or more years

1. Have you ever used cannabis for non-medicinal or recreational purposes?

Yes

No

*If “yes” to Q18, answer A and B below:*

1. Do you currently (within the last 3 months)use cannabis for non-medicinal or recreational purposes?

Yes

No

1. Do you intend to use cannabis for non-medicinal or recreational purposes in the future?

Yes

No

1. Have you ever used cannabis to manage your musculoskeletal pain (which includes muscle, tendon, bone or joint pain)?

Yes

No

*If “Yes” is selected on Q19, answer questions 20-35. If “No” is selected, skip to question 36.*

**Supplementary Table 2:**

1. Are you currently (within the last 3 months) using cannabis to manage your musculoskeletal pain (which includes muscle, tendon, bone or joint pain)?

Yes

No

1. Do you intend to use cannabis to manage your musculoskeletal pain (which includes muscle, tendon, bone or joint pain) in the future?

Yes

No

1. How long have or did you use cannabis to manage your musculoskeletal pain (which includes muscle, tendon, bone or joint pain)?

Less than 6 weeks

6 weeks to less than 3 months

3 months to less than 6 months

6 months to less than 1 year

1 year to less than 2 years

More than 2 years

1. What strain/type of cannabis do or did you use?

High-Tetrahydrocannabinol (THC) strain

Cannabidiol (CBD)

Nabilone

Levonantradol

Dronabinol

Nabiximols

A mix of the above

Unspecified/Conventional cannabis

I am not sure

1. How often do or did you use cannabis for the management of musculoskeletal pain (which includes muscle, tendon, bone or joint pain)?

Daily

Weekly

Monthly

Less than once a month

1. How much do or did you spend monthly on cannabis to manage musculoskeletal pain (which includes muscle, tendon, bone or joint pain)?

Less than $50

$50 to less than $100

$100 to less than $200

$200 to less than $400

More than $400

1. Why did you start using cannabis for the management of musculoskeletal pain (which includes muscle, tendon, bone or joint pain)? Please select all that apply.

Advised or recommended by a medical doctor

Advised by a health professional other than medical doctor

Advised by family member, friend, or another individual who is not a health professional

My pain was insufficiently controlled using other medications and/or treatments

I was interested in managing pain without conventional medications and/or treatments

I wanted to avoid side-effect(s) that I experienced using other medications and/or treatments

Other (please specify): _______________________

1. What is or was your preferred mode of cannabis use? Please select all that apply.

Smoking

Vaporizing

Oil

Tinctures

Capsules

Edibles (Brownies, Cookies, Gummies, etc.)

Topical application

Patch Formulation

Other method (Please specify):_______________________________

1. Where do or did you acquire your cannabis? Please select all that apply.

Friend/Relative/Someone I Know

Cannabis Dispensary/Compassionate club

Dealer/On the street

Homegrown, without license

Homegrown, with license

Health Canada licensed provider

1. In your opinion, how effective was or has cannabis been at controlling your musculoskeletal pain (which includes muscle, tendon, bone or joint pain)?

Very effective

Somewhat effective

Slightly effective

Not effective

1. In your opinion, how effective has cannabis been in controlling your musculoskeletal pain (which includes muscle, tendon, bone or joint pain) c*ompared* to prescription medication?

Much more effective

Somewhat more effective

No difference

Somewhat less effective

Much less effective

I have not used prescription medication to manage my musculoskeletal pain

1. Has your use of other pain medications changed since you started using cannabis? Please check all that apply.

Increase in other pain medication use

No change in other pain medication use

Decrease in other pain medication use

*If decreased, which ones: __________________________________________

*If increased, which ones: ___________________________________________

1. Have you experienced any of the following side-effects/negative effects from taking cannabis to manage musculoskeletal pain (which includes muscle, tendon, bone or joint pain)? Please select all that apply.

Dizziness

Dry mouth

Nausea

Fatigue

Lack of motivation

Depression

Vomiting

Weight gain

Euphoria

Other (please specify):_____________________________

None of the above

1. Have you ever used cannabis to treat a symptom/condition other than musculoskeletal pain?

For example: nausea, headache, sleep disturbances, low appetite/weight, anxiety, depression, post-traumatic stress disorder, etc.

No

Yes

1. If “yes” to question 33, which condition did you **first** use cannabis to manage?

Musculoskeletal pain (which includes muscle, tendon, bone or joint pain)

Another symptom or condition

1. Please select all of the symptoms/conditions other than musculoskeletal pain you have treated using cannabis. If none apply, select “none of the above”.

Nausea

Headache

Sleep disturbances

Appetite/weight

Anxiety

Depression

Post-Traumatic Stress Disorder (PTSD) Symptoms

Other (please specify): ____________________

None of the above

**Supplementary Table 3:**

1. Would you ever consider using cannabis to manage your musculoskeletal pain (which includes muscle, tendon, bone or joint pain)?

Yes

No

1. Please rate your agreement with the following statements:

|  | Completely Agree | Somewhat Agree | Neither Agree or Disagree | Somewhat Disagree | Completely  Disagree |
| --- | --- | --- | --- | --- | --- |
| There is a **stigma** associated with using cannabis | 🞎 | 🞎 | 🞎 | 🞎 | 🞎 |
| There is a potential for **side effects** from using cannabis | 🞎 | 🞎 | 🞎 | 🞎 | 🞎 |
| I do not know how to **access/obtain/purchase** cannabis to manage musculoskeletal pain | 🞎 | 🞎 | 🞎 | 🞎 | 🞎 |
| I do not know how to **use/take/administer** cannabis to manage musculoskeletal pain | 🞎 | 🞎 | 🞎 | 🞎 | 🞎 |
| I do not know **what type of cannabis product/formulation** to use to manage musculoskeletal pain | 🞎 | 🞎 | 🞎 | 🞎 | 🞎 |
| I am not **aware of any** **evidence to support** the use of cannabis to manage musculoskeletal pain | 🞎 | 🞎 | 🞎 | 🞎 | 🞎 |
| I would prefer to use **more conventional medications and/or treatments** to manage my musculoskeletal pain | 🞎 | 🞎 | 🞎 | 🞎 | 🞎 |
